# Integrated wafer-scale manufacturing of electron cryomicroscopy specimen supports

**DOI:** 10.1016/j.ultramic.2021.113396

**Published:** 2022-01

**Authors:** Katerina Naydenova, Christopher J. Russo

**Affiliations:** MRC Laboratory of Molecular Biology, Francis Crick Avenue, Cambridge CB2 0QH, UK

**Keywords:** Grid, HexAuFoil, TEM, cryoEM, CMOS, MEMS

## Abstract

We present a process for the manufacture of electron cryomicroscopy (cryoEM) specimen supports with an integrated foil–grid structure, using cryogenic vacuum evaporation (cryoEvap) and patterned electroplating on a silicon wafer substrate. The process is designed to produce a pattern of nanometre scale holes in a thin metal foil, which is attached to a pattern of micrometre scale grid bars that support it and allow handling of the millimetre scale device. All steps are carried out on a single 4 inch (100 mm) silicon wafer, without any need to handle individual grids during processing, and yield about 600 supports per wafer. The approach is generally applicable to the problem of creating a thin foil with nanometre scale features and a micrometre scale support structure; here it is used to make an all gold, HexAuFoil type design. It also allows for the addition of custom fiducial markers and patterns which aid in locating and identifying particular regions of a grid at several length scales: by eye, in an optical microscope, and in the electron microscope. Implemented at scale, this manufacturing process can supply ample grids to support the continued growth of cryoEM for determining the structure of biological molecules.

## Highlights

• A method for manufacturing integrated cryoEM specimen supports is presented.
• It is used to produce all gold, HexAuFoil type grids for motion-free imaging on 4 inch silicon wafers.
• Custom fiducial markers are included in the grid design.
• This method will allow scaled manufacture of supports to meet increasing demand.
• The supports made improve the quality and throughput of high resolution structures determined by cryoEM.

## 1 Introduction

Technology in specimen supports for cryoEM has advanced rapidly in the last 10 years [1], [2], just as detectors have become faster and more efficient [3], and electron microscopes have become more automated and easy to use [4], [5]. The latter creates more need for high-quality specimen supports, the production of which has struggled to keep pace with the exponentially increasing demand for structure determination by cryoEM.

At the advent of transmission electron microscopy (TEM), the need for a stable, conductive and versatile support for specimens was immediately clear. Early work used fine wires, or small pieces of fine metal mesh, to support the specimens under examination [6]. Later, techniques used to produce metal apertures for high energy particle physics experiments and grids for vacuum tube and photographic plate manufacture were adapted to make a flat metal mesh on a template by electroplating. Soon, the 3 mm metal grid became the world-wide standard specimen support, with the major microscope manufacturers, including Siemens, The Radio Corporation of America (RCA), Vickers, Philips, VG, Zeiss, Hitachi and Japan Electron Optics Laboratory Company (JEOL) all eventually producing specimen loading airlocks and holders compatible with it. Commensurate with demand, by the 1950s there were several manufacturers in the US, Europe and Japan all making grids for the burgeoning electron microscopy market. These included Jelliff Manufacturing Co. (Southport, CT USA) who produced 3 mm grids under the brand Lectromesh, RCA (New York) using their own brand, Smethurst High-Ligh Ltd (Bolton, Lancashire UK) under the Athene brand, and Okenshoji Co., Ltd (Tokyo, Japan) under their brand, to name a few. Later, many more companies entered the market including Mason and Morton, Gilder and Graticules Optics (Maxtaform) in the UK alone, with a variety of additional brands created by microscopy supply companies like Ted Pella, EMS, SPI and Agar and manufactured under contract by various suppliers. So it seems something of a historical accident of highly successful commercialisation and wide distribution of these inexpensive grids, that the techniques for making them have been lost to most microscopy research labs. This is in stark contrast to thin support foils/films, which were introduced as an additional, electron transparent support on top of the metal grids [7], [8], [9]; it was only these, which make intimate contact with the specimen, that were and still are routinely made by investigators themselves, using simple vacuum coating instruments [10].

As micro-lithography techniques became widespread with the growth of the semiconductor industry, foils with well defined arrays of holes were created to improve the reliability and regularity of the structure of the thin foil suspended across the ubiquitous 3 mm metallic grid. One method currently in common use relies on creating a uniformly sized array of holes in a polymer template [11]. The hole pattern is then replicated by a carbon film, deposited by high temperature sublimation in vacuum onto the sacrificial template, and transferred onto a metal 3 mm grid. These supports are widely used for cryoEM and are commercially available under the name Quantifoil. An alternative, yet similar support, dubbed C-flat, is produced by using a hard template and a soluble release layer to replicate a well defined, holey pattern onto a carbon film [12]. These have the advantage of having a more uniform carbon thickness and quality but are more fragile to handle. A third method of foil patterning described recently uses a poly(dimethylsiloxane) (PDMS) stamp to produce carbon films with a regular array of holes as small as 500 nm [13]. The need for *carbon* foils at all was called into question with the design and implementation of a patterned gold foil on a gold grid, called UltrAuFoil [14]. These all-gold supports have improved mechanical stability during cryoplunging and reduced charging and movement under electron irradiation at cryogenic temperatures [15], all of which are highly desirable, particularly as the resolution of most cryoEM reconstructions extended to below 4 Å. Notably, both recent structures of apoferritin in the range of 1.2 Å resolution used specimen supports of this type [16], [17].

To date, all of these methods involve the separate production of the grid and the foil, and the handling of individual grids at at least one, and sometimes at several stages of manufacture. Other specimen supports have been created with silicon/silicon nitride windows, which were previously developed for X-ray microscopy [18] and later for producing single solid state nanopores [19], or silicon carbide substrates [20]. However, these supports are less suitable for typical cryoEM since the suspended foils are poor conductors and have other difficulties in handling and imaging under cryogenic conditions.

To keep up with the ever increasing demand for specimen supports for cryoEM, a new approach to the manufacture of the foil and the grid as one unit device was clearly needed. So we developed a fully integrated, on-wafer process for the large scale manufacture of specimen supports for cryoEM, in the model of manufacturing semiconductor devices. Importantly, the process does not rely on prefabricated grids and is fully scalable using standard, commercially available machines for semiconductor processing. We have created a process based on 4 inch (100 mm) wafers here, which yields 594 grids/wafer, but can be scaled for manufacture on 6, 8 or 12 inch wafers with appropriate tooling. This would increase the yield, in proportion to the area of the wafer, to approximately 1500, 2500 or 6000 respectively.

We recently described how an optimised all-gold specimen support design, dubbed HexAuFoil, eliminates specimen movement during cryoEM imaging [21]. The HexAuFoil specimen supports have already been used to determine several structures of biological interest, including the SARS-CoV-2 RNA polymerase in the presence of the drug favipiravir [22] and the light harvesting complex 2 from a purple bacterium [23]. The key parameter in this grid design is the aspect ratio of the foil thickness to the foil hole diameter. We experimentally demonstrated that an optimal ratio is around 1:10. A typical single-particle cryoEM specimen thickness of not more than 300 Å necessitates a similar foil thickness, which in turn entails a hole size of 300 nm. For smaller specimens in thinner ice, the optimal hole diameter is even smaller. These optimal hole diameters are smaller than what is attainable with the previous grid manufacturing methods, where the smallest feature size is determined by the diffraction limit set by the wavelength of the light used for patterning. To make the HexAuFoil specimen supports, with their 200–300 nm sized holes, a new manufacturing method was required, and was described previously in brief [21]. Here we incorporate this method for making gold foils with small holes into a multi-mask process to make complete cryoEM specimen supports without any transfer steps or need to handle grids individually.

## 2 Materials and methods

**Fig. 1:**
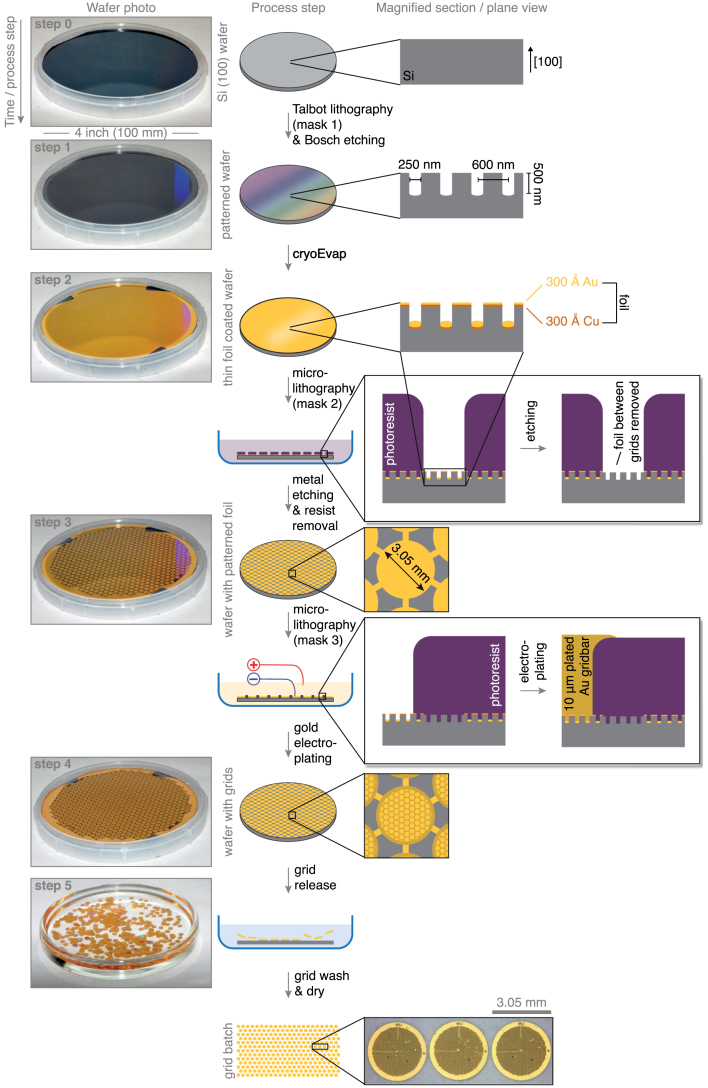
**Wafer-scale process for manufacturing of all-gold specimen supports for cryoEM.** The flowchart summarises the integrated wafer-scale process for grid manufacturing. Photographs of a wafer during the five stages of the process (as numbered in the Materials and Methods), and of the released grid batch in water, are shown on the left. The photograph in the lower right shows three grids (with the grid bar side facing up) from a batch produced by this process.

The complete process requires three lithography steps, an electron-beam evaporation with a cryogenically cooled wafer stage, an electroplating step, and several dry and wet chemical etches, all performed on a single wafer. Below, we briefly describe the process stages, which are also outlined in Fig. 1. The detailed procedure is provided in Appendix A, the required equipment and materials are listed in Table A.1, some troubleshooting advice is given in Table A.2, and the mask designs are available in the Supplementary Material.

### 2.1 Wafer patterning

We begin with a bare 4 inch silicon (100) wafer. First, a regular array of holes (cylindrical wells) needs to be created across the whole area of the wafer. For the sub-diffraction limit sized HexAuFoil holes (100 to 400 nm in diameter), this pattern is made by phase interference (Talbot displacement) lithography with an ultraviolet source [24], [25]. Electron beam lithography or focused ion beam milling could, in principle, be used to generate the high resolution features required for the hole pattern array, but these techniques are inherently serial and do not scale to the number of units required ($>10^{6}$ holes per grid × $10^{6}$ grids per year). For larger holes, the pattern could be made by conventional photolithography. The hole pattern in the photoresist is then etched into the silicon by deep reactive ion etching, to a depth approximately equal to twice the hole diameter, and with 90${}^{\circ }$ sidewalls [26], [27]. The depth-to-width ratio of the holes and the sidewall sharpness are crucial parameters which ensure that the subsequently deposited metals in the bottom of the wells are not contiguous with the top surface foil. For these experiments, this lithography step was carried out by Eulitha AG, in Switzerland.

### 2.2 Thin foil deposition

The second part of the process replicates the hole pattern in gold by cryogenic evaporation. Specifically, we deposit a thin sacrificial copper layer onto the silicon wafer, followed by a gold layer, each being 300 ± 10 Å thick (Fig. A.2). Both layers are deposited by electron beam evaporation, with the substrate (wafer) cooled with liquid nitrogen to 80–90 K. We named this process cryoEvap. The low temperature limits the diffusion rate of deposited adatoms on the surface, thereby creating smaller grains within the metal layer. This in turn allows us to make the foil thinner (300 Å) while still continuous, and to replicate the round contours of the holes more precisely. The typical mean linear intercept grain size of the cryo-evaporated gold film is 10 nm; this sets the minimal thickness of a stable foil and the roundness of each hole.

### 2.3 Grid outline

Third, we outline the contours of each grid. The edges of the individual grids are defined directly on the patterned foil by conventional photolithography, with a positive photoresist layer. This mask step is used to remove nearly all the metal foil from the regions in between the grid outlines by wet etching, after which the resist is removed. Only thin strips of foil connecting each grid area to its neighbours are preserved. This is important to maintain electrical contact between regions during subsequent electroplating.

### 2.4 Grid bar deposition

**Fig. 2:**
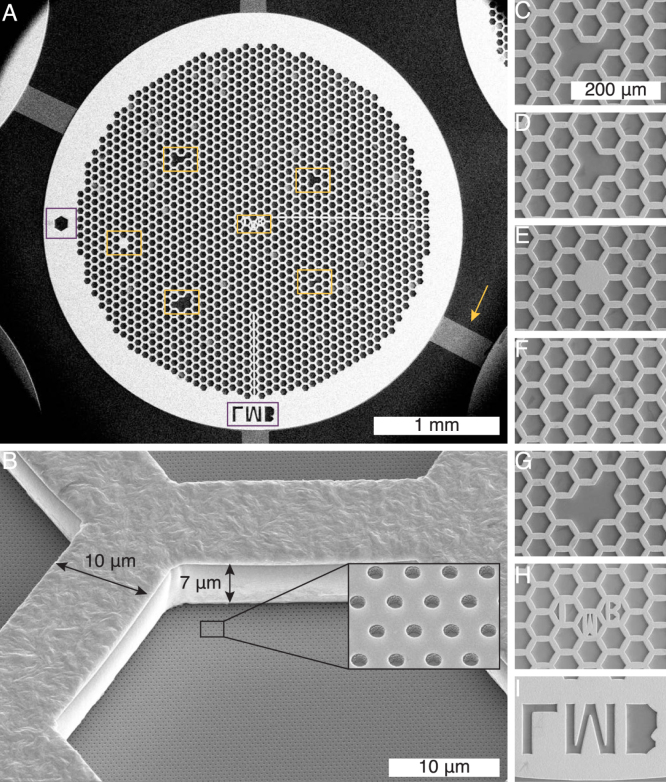
**On-wafer HexAuFoil grids.** Scanning electron micrographs of a HexAuFoil grid, fully fabricated on a silicon wafer, and still attached to the wafer, are shown. The samples were irradiated with 30 keV electrons, and imaged on an Everhart–Thornley detector for collection of secondary electrons. **(A)** One full HexAuFoil grid has a diameter of 3 mm and is separated from the neighbouring grids on the wafer. The darkest areas correspond to the exposed silicon surface of the templating wafer. The *yellow boxes* outline the six fiducial markers on the grid, which label each of the four quadrants. These fiducials are magnified in panels **(C–H)**. The grid also has two rim marks (*purple boxes*), which are visible by eye (requiring dimensions of at least 0.2 mm), and one of them is shown in panel **(I)**. The largest quadrant mark **(G)** is clear of foil, and this location can be conveniently used to perform electron microscope alignments and flux measurement. The *yellow arrow* points to a thin gold foil connection strip between two grids which provides continuous electrical contact for electroplating. **(B)** Each hexagon is 50μm wide, and contains 6000–7000 holes. The grid bars are formed of electroplated gold, and are 10μm wide and 7μm thick in this example. The micrograph is recorded at 30${}^{\circ }$ tilt. The *inset* shows the holey foil in the boxed area, magnified 7×. The writing in panels **(A, H, I)** is mirrored by design; when the grid is separated from the wafer and viewed from the flat foil side, it reads ‘LMB’ correctly.

The fourth part of the process adds grid bars directly onto the foil. The lateral edges of the grid bars are also established by photolithography, using another mask and positive resist layer. The grid bars are then deposited onto the foil by electroplating, where the electrical conductivity across the wafer is important to ensure uniform deposition of the metal. We used a gold sulphite/thiosulphite electrolyte solution, for which the optimal current density is 2–10 mA/cm${}^{2}$ at 54 °C. Under these conditions, using the patterned wafer as a cathode and a platinum plate anode, we deposited 7μm thick grid bars and rims in just under 1 h. The grid bar thickness can be controlled by adjusting the electroplating duration, provided the patterned photoresist layer is sufficiently thick. Uniformly thicker photoresist layers, if required, can be obtained by double-coating the wafer with resist, or by using a different photoresist compatible with the electroplating step. Once the grid bar thickness exceeds that of the photoresist, the gold deposition continues isotropically from the top surface, forming a rounded cap on the grid bar, which may obscure part of the holey foil area. At the end of this step, the grid is complete but remains attached to the silicon wafer via the copper adhesion layer (Fig. 2).

### 2.5 Grid release

**Fig. 3:**
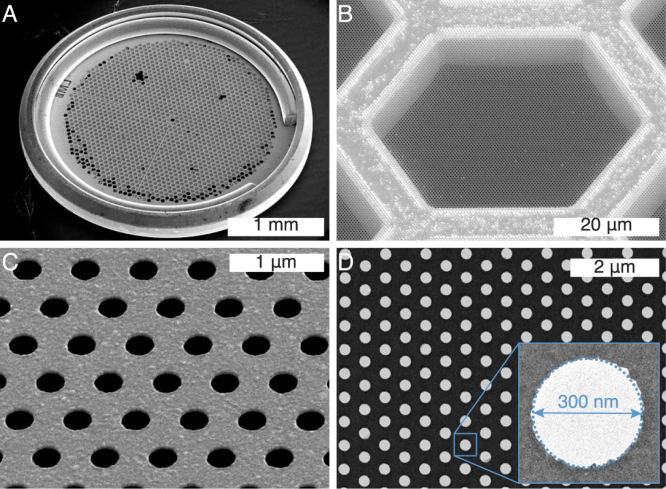
**HexAuFoil grids released from the wafer post-fabrication.(A)** Scanning electron micrograph (45${}^{\circ }$ tilt, with 2 kV acceleration voltage using an Everhart–Thornley detector) of the bar side of the grid after release from the wafer and removal of the copper adhesion layer. The grid is clipped in a standard clip-ring/C-clip holder used for transmission electron microscopy. **(B)** Scanning electron micrograph (45${}^{\circ }$ tilt, with 30 kV acceleration voltage using an Everhart–Thornley detector) micrograph of one of the hexagons on the same grid, acquired from the flat side of the grid, i.e. after the grid is flipped over relative to its orientation in **(A)**. This is the side of the grid that was originally covered with the sacrificial copper layer, making contact to the silicon template. The holey foil spans each grid hexagon and remains intact. **(C)** Scanning electron micrograph (45${}^{\circ }$ tilt, with 30 kV acceleration voltage using an Everhart–Thornley detector) micrograph of the holey gold foil demonstrates the edge flatness of each hole and surface flatness of the foil. These characteristics are important for the formation of a thin, flat ice layer when the grids are used for cryoEM sample preparation. **(D)** Transmission electron micrograph (120 keV) of the suspended gold foil on the grid after release from the wafer. The spacing between the hole centres is 600.00 nm. The *blue box* delineates the area magnified in the *inset*. The hole diameter is 300 nm as indicated. The edge roughness is limited by the grain size of the gold foil, in this case approximately 10 nm for gold deposited at 85–90 K substrate temperature. The *dashed blue circle* is for comparison with the roundness of the hole.

In the final part of the process, the grids are separated from the wafer, and the copper is removed (Fig. 3) The grids are separated from the wafer by dissolving the silicon in hot (80 °C) potassium hydroxide. The grids lift off the wafer almost instantly on immersion into the KOH solution, which also removes the residual photoresist. The thin interconnecting foil strips between individual grids break spontaneously during the liftoff, leaving the individual grids floating freely in the solution. The copper layer attached to the gold foil is then removed from the released grids with a piranha solution (3 H${}_{2}$SO${}_{4}$ : 1 H${}_{2}$O${}_{2}$), which also eliminates any remaining organic surface contaminants from the photolithography steps. The batch of grids can be kept in water or dried and stored in air for distribution and use.

## 3 Results and discussion

**Fig. A.1:**
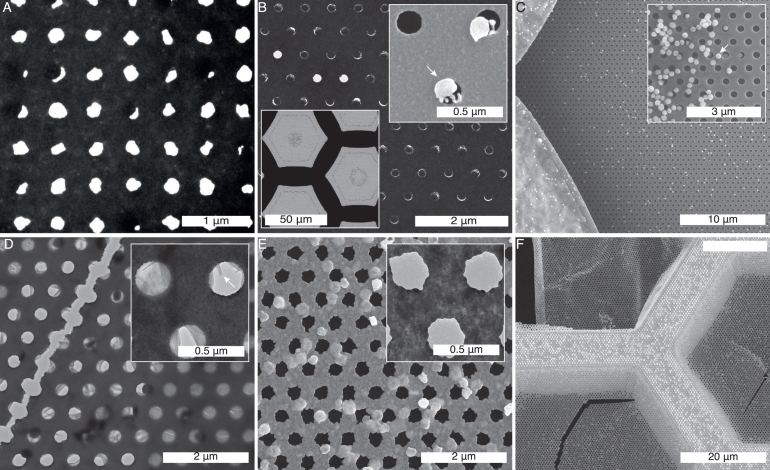
**Possible defects due to incorrect processing during wafer-scale grid manufacturing.** All transmission electron micrographs are acquired at 120 kV acceleration voltage on a phosphor-coupled CCD, and all scanning electron micrographs are at 30 kV on an Everhart–Thornley detector. **(A)** Transmission electron micrograph of gold foil with ∼100 nm grain size, fabricated at room temperature. The relatively large grain size prevents the formation of round holes in the foil. **(B)** Transmission electron micrograph of gold foil, where most of the holes are blocked by residual gold. The *lower inset* is a low-magnification transmission electron micrograph of the same grid, which shows that most of the blocked holes are located near the centres and the edges of the grid hexagons. This is probably related to the speed of the grid release in these regions. The *upper inset* shows a scanning electron micrograph of the same foil, acquired with the flat side of the grid facing the detector at 0${}^{\circ }$ tilt. This shows that the holes are blocked by discs of gold, carried over from the bottom of the wells in the template wafer. **(C)** Scanning electron micrographs of a grid after electroplating and resist removal, while still attached to the templating wafer. The small gold crystals (*arrow* in the *inset*) originate from the electroplating process, and tend to cluster near grid bars. **(D)** Transmission electron micrographs of a grid after KOH-assisted release from the template wafer. The holes are obstructed by residual Si crystals (*arrow* in the *inset*) still attached to the foil. **(E)** Scanning electron micrograph of a grid which was treated with hot (∼100 °C) piranha solution. This etched the thin gold foil, resulting in enlarged diameter and irregular shapes of the holes. The *inset* shows a transmission electron micrograph of the same grid. **(F)** Scanning electron micrograph of the flat side of a grid, at 45${}^{\circ }$ tilt. This grid was released from the templating wafer by etching the sacrificial copper layer in hot piranha solution, rather than by KOH-assisted Si etching. This release method is less reliable and slower. It results in partial etching of the holes as in **(E)**, but also causes many of the suspended foils to break, making them unsuitable for high-resolution imaging.

**Fig. A.2:**
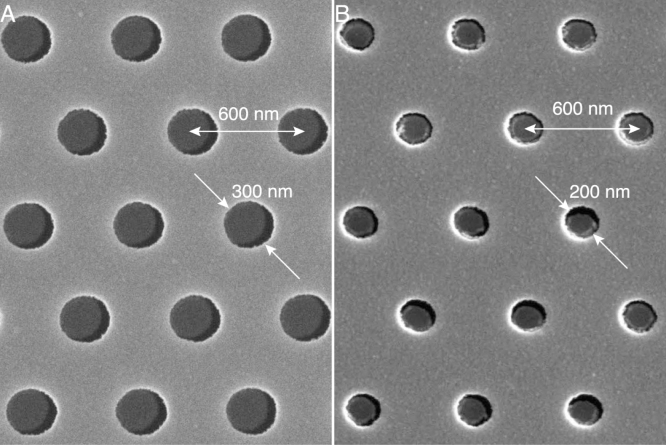
**HexAuFoil grids with 200–300 nm hole diameters.** Panels **(A)** and **(B)** show scanning electron micrographs of two HexAuFoil gold foils, still attached to the templating silicon holey wafer via the sacrificial copper underlayer. Both micrographs are acquired at 0${}^{\circ }$ tilt, with 30 kV acceleration voltage using an Everhart–Thornley detector. The scale is set by the centre-to-centre hole spacing, which is 600 nm for both. The foils in panels **(A)** and **(B)** differ only by the templating hole diameter, which is 300 nm for **(A)** and 200 nm for **(B)**, respectively. Discs of gold foil can be seen at the bottom of each hole in the silicon wafer, with a shadowing angle dependent on the viewing angle from the gold source during electron beam evaporation of the foil towards the given point on the wafer. If the holes are insufficiently deep, these discs can remain attached to the foil and obstruct the holes when the foil is released from the wafer. We found that a depth of 500 nm is sufficient to avoid this for 200–300 nm holes and 300 Å thick copper and gold foils.

The scaleable process described here allows around 600 grids to be made per 4 inch wafer. Processing of one wafer takes approximately 3 days (Appendix A), and can be easily parallelised by processing multiple wafers per batch. Crucially, none of the steps in this process require handling of individual grids. The released grids can be stored in batch form, but could also be packaged individually. The techniques and equipment used here, save the cryoEvap step, are all common in foundries and fabrication laboratories (Table A.1). A key advance to produce nanoscale features of sufficient quality was the development of cryoEvap as a method for creating gold foils with fine grain structure (Fig. A.1A). The process can be scaled up to 12 inch wafers without modification, thus yielding ten times more grids per wafer with no additional labour. This is in contrast to the current, labour-intensive float methods for grid manufacturing, where each individual grid needs to be handled manually multiple times.

Many micro-electromechanical system (MEMS) type fabrication processes, including this grid fabrication method, require a final step to separate the formed functional devices from the surface of the wafer. This is usually achieved by dry or wet etching of the supporting wafer, grinding, dicing, or a combination of these [28], [29], [30]. After testing many potential methods, we chose to release the fully formed grids from the wafer by chemical etching of the silicon (Fig. A.1B–F). In principle, other methods which allow for the silicon wafer to be re-used can be envisioned. In practice, however, we obtained the most reproducible, contamination-free results by dissolving the silicon. It might also be possible to design a release method, which keeps the grids loosely attached to each other in a liftoff procedure after separation from the backing wafer [31]. This has the potential to further simplify handling during grid packaging in a commercial setting.

**Fig. 4:**
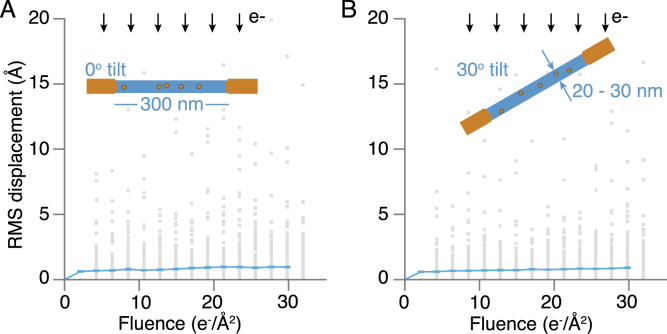
**HexAuFoil grids produced by the integrated wafer-scale method eliminate cryoEM specimen movement during electron irradiation.** In-plane **(A)** and 30${}^{\circ }$ tilt **(B)** movement statistics of gold nanoparticles in the HexAuFoil grids produced by the wafer-scale method demonstrate the performance of these grids in terms of reducing specimen movement is equivalent to that of the HexAuFoil grids produced by the previously described small scale method [21]. The *grey points* indicate the displacements of individual tracked particles, the *blue lines* correspond to mean values, and the error bars indicate standard deviations.

We examined the performance of the grids, manufactured by the procedure described above, and verified that it is robust during cryo-plunging and clipping in standard TEM holders (Fig. 3A, Appendix B). We also confirmed that HexAuFoil grids made using this process eliminate specimen movement (Fig. 4), as previously reported in [21], where the grids were manufactured by a float transfer method. The foil side of the grids, which is the side facing the wafer during the fabrication process, is completely flat and in tension. As a result, blotting conditions for cryo-plunging may need to be adjusted relative to those used for other grids; however, we found no differences were needed using a manual plunger or the Vitrobot Mk. 4 (FEI). In principle, the residual stress in the foil can be adjusted by varying the cryo-evaporation temperature and rate, and the electroplating conditions (temperature, agitation, and current density). In the conditions of the procedure described here, we expect that the evaporated thin gold foil is under moderate tensile stress [32], whereas the electroplated gold grid bars are under slight compressive stress [33]. Thus, when the grids are released from the substrate, the foil is suspended across the grid bars, in tension.

We have incorporated some features into the grid mask design to allow easy and unambiguous orientation determination of the grid, both in the electron microscope and under observation with the unaided eye. Two marks on the rim of the grid (‘LMB’ or ’MRC’ and a hexagonal opening), as well as the large opening in the foil (Fig. 2) are visible by eye. The large opening provides an area which can be used for beam alignments in the electron microscope. The two lines (longer and shorter) intersecting the grid hexagons, which can be seen in the TEM, point in the direction of the two rim marks. In addition, each quadrant of the grid (defined by the intersection of these two lines) contains a unique fiducial, identifying the quadrant. These fiducial marks, and the letters in the centre mark, break the symmetry of the grid pattern, and allow unambiguous identification of every hexagon on the grid. This allows the orientation of the grid in the electron microscope to be determined, which can then be correlated with its orientation during specimen preparation or other microscopy steps.

In this design, the grid hexagons are 50μm wide, and the grid bars are 10μm × 7μm in cross-section. This geometry is compatible with automated cryoEM data collection software with minor modifications to search for a hexagonal, rather than a square array pattern. The grid bar pattern, width, spacing, and all the alignment features, can be modified freely using the mask designs provided in the supplementary materials. For example, supports with variable mesh spacing can be created across a single grid or other fiducial marks identifying each grid could be added.

A side benefit of this grid design is presence of intrinsic reference structures which can be used to calibrate magnification. The regular spacing of the holes in the foil (600 nm hole to hole pitch, for the templates used in this work), is set by phase-interference lithography on the silicon template, and can be used to accurately (to better than 1 part in 10,000) calibrate low magnifications in the TEM. At the same time, high magnifications (<1.5 Å/pixel) can be calibrated using the Au (111) lattice reflections at 1/2.347 Å resolution (at 80 K temperature).

## 4 Conclusions

We demonstrated an integrated, wafer-scale process for the production of HexAuFoil grids, which have holes smaller than 300 nm. In principle, the grid fabrication method described here can be extended for grids with larger, micron-sized holes, more complicated hole patterns, and/or thicker foils if required for the particular application. In cases when nanoscale features are less critical, cryoEvap could be replaced with conventional electron beam or thermal evaporation, and the holes can either be pre-patterned on the Si substrate as above, or patterned in the foil after deposition by conventional photolithography and wet etching. This wafer-scale grid fabrication process can be adapted to incorporate other specimen support technologies. For example, nano-wires could be grown on the grid bars for self-wicking grids [34], or a continuous graphene monolayer could be added to the flat side of the grid [35], [36], [37], but might require modification of the release process. The same wafer-scale process could also be adapted to produce grids optimised for applications other than single-particle cryoEM, like high pressure freezing and electron cryotomography.

## Declaration of Competing Interest

The authors are inventors on a patent application (GB2004272.7) on the design and fabrication of specimen supports, filed by the Medical Research Council as part of United Kingdom Research and Innovation.

## Figures and Tables

**Table A.1:** **Equipment and reagents.** The required instruments, tools and reagents for the process (fully described in the appendix) are listed, with suppliers.

Category	Item	Supplier
**Reagents &**	4” Si (100) wafer with template hole pattern	Eulitha AG
**Materials**	(p-type, Boron-doped, 0.5 mm thick, 1–30 Ωcm)	
	Copper pellets for evaporation, 99.999%	KJ Lesker
	Gold pellets for evaporation, 99.999%	KJ Lesker
	Wafer clip-on holder (OFHC copper)	Made in-house
	5” masks (2×)	Compugraphics
	Megaposit SPR 220-7.0 photoresist	Rohm and Haas Electronic Materials
	Microposit MF26A developer	Shipley Europe
	Microposit 1165 remover	Dow Electronic Materials
	Gold etchant TFA	Transene
	MetGold ECF 33B electroplating solution	Metalor Technologies SA
	Acetone (CMOS grade)	Alfa Aesar
	Isopropanol (CMOS grade)	Alfa Aesar
	Potassium hydroxide pellets 99.99%	Sigma
	Sulphuric acid 99.999%	Sigma
	Hydrogen peroxide 34.5–36.5%	Sigma
	H${}_{2}$O (18 MΩ filtered)	Millipore

**Glassware &**	Borosilicate crystallising dishes	Duran
**Tools**	(5 × 115 mm and 5 × 190 mm)	
	Borosilicate beakers	Duran
	(200 mL and 25 mL)	
	Borosilicate petri dish (2 × 200 mm)	Duran
	10 mL glass test tubes	Fisher Scientific
	Wafer tweezers, stainless steel	Idealtek
	Wafer tweezers, carbon tipped	Idealtek
	Ceramic-tipped fine tweezers	Idealtek
	Fine-tipped, negative-action tweezers	Dumont
	Magnetic stir bar	Fisher Scientific
	Aluminium foil	Prowrap
	Lint-free wipes	Texwipe
	Nitrogen gun	PCL
	Timer	VWR
	Filter paper (190 mm ⌀)	Whatman
	Support mesh and clamp for filter paper	Made in-house
	(316 stainless steel)	

**Instruments**	E-beam cryo-evaporator	Moorfield
	UV ozone cleaner	Jelight Co Inc
	Spinner	Laurell Technologies Co
	Hotplate	Techne
	Mask aligner	Quintel
	Upright optical microscope (5×- 50×	Zeiss
	air objectives, reflection illumination)	
	Vented oven	Binder
	Electroplating equipment set	Yamamoto
	Weighing scale	Mettler Toledo
	Hotplate/magnetic stirrer	IKA

**Table A.2:** **Troubleshooting.** Possible problems occurring during the procedure and troubleshooting advice can be found in the table. The wafer/grids can be imaged by optical and/or scanning electron microscopy after every step of the process to simplify troubleshooting.

Step	Problem	Possible reason	Solution
2–5	Foil grain size is larger than	Evaporation temperature	Make sure the template
	20 nm, compromising the	and/or deposition rate were	is at liquid nitrogen temperature
	roundness of the holes.	too high.	before starting the evaporation
			of each layer; measure rate
			with calibrated crystal thickness
			monitor, and do not exceed 1 Å/s.
			
1–5	A large fraction of the foil	Evaporation onto a	Ensure the holes in the silicon
	holes are obstructed.	template with insufficiently	template are at least 400 nm deep
		deep or contaminated holes.	and free of contaminants,
			i.e. clean with piranha and/or
			aqua regia prior to use.
			
25–30	Grids are contaminated	Deposition during	Perform regular maintenance
	with spherical, sub-1μm	electroplating.	of electroplating solution and
	gold crystals, especially		equipment, including fresh
	clustering near/on grid bars.		chemicals and/or filtration;
			ensure current density is optimal.
			
31–33	Foil is contaminated with	Residual silicon on	Repeat KOH etch, make sure
	electron dense, rod-shaped	backside of foil.	temperature and concentration
	crystals.		are correct.
			
35	Foil holes are etched	Hot piranha solution	Make sure piranha solution
	to enlarged, irregular shapes.	attacks the thin gold film.	is cooled to room temperature
			prior to use.

**Table A.3:** **Surface treatment of HexAuFoil specimen supports.** Typical settings for making HexAuFoil grids hydrophilic by using plasma cleaning or residual air glow discharge are given.

Parameter	Fischione 1070	Edwards S150B	TedPella easiGlow
Atmosphere	9:1 Ar:O${}_{2}$	Residual air	Residual air
Process pressure	21 mTorr	150 mTorr	0.39 mBar (290 mTorr)
Power/Current	40 W	30 mA	25 mA
Exposure time	120–180 s	60 s	90 s

**Table A.4:** **Vitrification by plunge freezing.** Typical settings for HexAuFoil grid vitrification using different plunge freezing instruments are given.

Parameter	Manual plunger	Vitrobot Mk. 4	Leica GP2
Temperature	4 °C	4 °C	4 °C
Relative humidity	100%	100%	100%
Wait time	0 s	0 s	0 s
Force setting	n/a	10	n/a
Blot time	15 s	5 s	5 s
Drain time	0 s	0 s	0 s

## Appendix A Detailed procedure

### Warning

This procedure involves hazardous chemicals and cryogens. It is intended for the use by experienced scientists and engineers who know how to handle such chemicals and instruments, and have completed all appropriate local safety training and risk assessments.

### Evaporation (Day 1)

*Time:* ∼ *0.5 days*

1. Clean the wafer with 2 min of UV ozone plasma. Blow away any particulates from its surface, using the nitrogen gun. Clip the wafer in the evaporator holder plate.
2. Load the wafer into the evaporator. Make sure the copper holder plate is in tight contact with the cryostage. When the chamber pressure falls below 8$\times 10^{-8}$ mBar, start cooling the cryostage with liquid nitrogen. Wait until the temperature stabilises at around 80 K and the vacuum at 2$\times 10^{-8}$ mBar.
3. With the sample shutter closed, and the electron beam shutter open, warm up the copper source with the electron beam (9 kV, 30–50 mA) until a stable evaporation rate of 0.8–1 Å/s is achieved. Open the sample shutter, evaporate 300 Å of copper, and close the electron beam shutter when the target thickness is reached. Record the actual thickness on the crystal thickness monitor and close the sample shutter.
4. Top up the liquid nitrogen if necessary and wait for base pressure.
5. Warm up the gold source with the electron beam. In the same way as above, evaporate 300 Å of gold with 9 kV acceleration voltage at 40–60 mA current to achieve a rate of 0.8–1 Å/s.
6. Unload the wafer into the loadlock and let it slowly warm up to room temperature overnight under vacuum.

### Grid definition by lithography (Day 2)

*Time:* ∼ *1 day*

*Note:* The photolithography is carried out based on the photoresist manufacturer’s recommended protocols for 4” wafers, and parameters such as spin speed, exposure time and energy dose, pre- and post-exposure bake time and temperature, resist development and removal conditions may need to be adjusted according to the selected photoresist, wafer diameter and lithography equipment.

7. Preparation: 30 min in advance, take the photoresist out of the fridge, set the oven to 85 °C, the hotplate to 99 °C, and switch on the UV lamp and mask aligner. Switch to UV-filtered lighting in the room — all lithography steps need to be carried out under controlled lighting to avoid pre-exposure of the photoresist. Set up the mask aligner for 35 s exposure time in vacuum contact printing mode.
8. Check the first mask for defects, wear, or contaminants under the optical microscope, and clean with acetone if necessary. Place the mask (chrome side down) in the mask aligner and clamp by vacuum. This is the mask that establishes the outlines of the grids.
9. Clean any dust from the wafer using the nitrogen gun. Clean with UV ozone for 2 min.
10. Pour ∼10 mL of photoresist in a round-bottom glass tube. Attach the wafer to the spinner, dispense the resist while static, and bring up to 2000 rpm for 1 min. This should give an approx. 7μm thick layer. The spin speed is chosen based on the photoresist manufacturer’s recommended protocols but may need to be adjusted empirically to give the desired thickness.
11. Transfer the wafer to the hotplate for a 180 s pre-exposure bake. At the end of this, transfer it to the aligner.
12. Load the wafer and reposition the mask/wafer if necessary. Bring the mask into firm contact with the wafer (>5” Hg on our instrument, this may need to be adjusted as appropriate for other mask aligners). Expose for 35 s. This provides a total energy dose of 700 mJ/cm${}^{2}$ measured at 365 nm, as per the manufacturer’s recommendations for this photoresist and thickness, and may need to be adjusted accordingly for different light sources/and or resists.
13. Wait for ∼100 min to allow water (which is necessary to complete the photo reaction) to diffuse back in the photoresist film.
14. In the meantime, prepare two developer baths (in 120 mm ⌀ dishes) and three deionised water baths (190 mm ⌀).
15. At the end of the waiting time, transfer the wafer to the hotplate for a 135 s soft bake.
16. Gently lower the wafer into the first developer and gently swirl for 180 s. Move to the second developer and gently agitate until the wisps of resist stop coming off the wafer, plus 30 s more (this should be around 180 s again). Record time in developer.
17. Transfer through the three water washes, and cascade rinse with deionised water.
18. Blow dry with the nitrogen gun until completely dry. Hard bake at 85 °C for 20–25 min in the ventilated oven.
19. In the meantime, prepare a 120 mm ⌀ dish with gold etchant (TFA) and three deionised water baths as above.
20. Immerse the wafer in the etchant for 35 s with careful swirling to accelerate the etch. The TFA etch rate for gold is 28 Å/s at 25 °C, and the rate for copper is similar.
21. Move the wafer through the three water baths, and cascade rinse with deionised water.
22. Set up two baths of resist remover: one at room temperature (120 mm ⌀), and one at 80 °C on a hotplate (in 190 mm ⌀ dish). Immerse the wafer for at least 10 min in each bath, agitating gently.
23. Cascade rinse with acetone, then isopropanol, then water. Blow dry with nitrogen.
24. Repeat the positive resist lithography with the second mask up to the hard bake included (steps 8–18). This establishes the outlines of the grid bars and rims. Use the microscope on the mask aligner to accurately align the features of the second mask onto the pattern on the wafer from the first mask. At the end of the second lithography process, the wafer can be stored overnight in the dark, under dry nitrogen atmosphere.

### Electroplating (Day 3)

*Time:* ∼ *0.5 days*

25. Pour the electroplating solution in the electroplating bath, start circulating the solution, and heat to 54 °C. Insert the platinum anode into the bath.
26. Clean the wafer with UV ozone for 2 min. Clip the wafer into the cathode holder and heat in the oven at 54 °C for 15 min. The wafer must be slowly warmed to the process temperature to prevent the foil from delaminating upon insertion into the heated plating solution.
27. Insert the cathode (with the wafer) into the bath, and attach the negative terminal of the power supply to it. Make sure no bubbles are formed on the surface of the wafer. Make sure the positive terminal is attached to the platinum anode.
28. Set the agitator to 40 strokes/min (which corresponds to 7.7 m/min at 4” stroke).
29. Increase the voltage to around 0.8–0.9 V and keep adjusting to maintain a constant current of 0.13 A. For a larger or smaller plating area, adjust the current accordingly to maintain the same current density. Do not exceed the standard potential of water (1.23 V). The plating speed at this current density is ∼0.2μm/min. Run the current for 50 min to get a 10μm thick deposit for the grid bars, or adjust the time accordingly for the desired thickness. The current can be monitored in real time and stopped automatically when or just after the grid bars become level with the resist, if desired. The target current may need to be adjusted according to the optimal current density for the recommended plating solution (2–10 mA/cm${}^{2}$) and the total area of the pattern for electroplating.
30. At the end of the electroplating cycle, take out the cathode (with the wafer) into warm (50–60 °C) water and rinse well to remove residual gold plating solution.

### Grid release (Day 3)

*Time:* ∼ *0.5 days*

31. Set up a 300 mL 30% KOH bath at 80 °C with stirring. Place in a secondary container to prevent spills.
32. Immerse the wafer in the bath and agitate until all grids are released.
33. Remove the wafer from the bath. The wafer can be discarded now. Carefully pour out as much KOH as possible into waste and top up with clean 30% KOH solution. Leave at 80 °C for at least 10 min, swirling gently.
34. Carefully pour out as much of the solution as possible again and top up with 0.5 L clean water at room temperature. Swirl gently and repeat. Decant as much water as possible.
35. Pour in 100 mL piranha solution (3 H${}_{2}$SO${}_{4}$ : 1 H${}_{2}$O${}_{2}$, prepared ∼1 hr in advance so that it is cooled to room temperature) into the container with the grids and leave at room temperature for 5 min, swirling gently.
36. Carefully decant as much piranha solution as possible into waste and top up with 0.5 L clean water at room temperature. Swirl gently and repeat.
37. Grids can be stored under water until use or dried for packaging. If dry storage is desired, carefully decant as much of the water as possible. Pour out the remaining water with the grids (help with water sprayer bottle if needed) onto a filter paper held down with a metal ring weight on a metal support mesh in a clean container. Move the grids so that they are not overlapping each other. Take out the paper with the support mesh and let dry in clean air or nitrogen.
38. Optically inspect the grids for any residue, if necessary repeat KOH and/or piranha cleaning, and washes. Table A.2 helps with troubleshooting.

## Appendix B Usage notes for cryo-EM

HexAuFoil grids, produced by the integrated wafer-scale process, can be used for specimen preparation and structure determination by cryoEM much like other widely used specimen supports with carbon or gold foils. We follow standard methods for grid handling, surface treatment, and vitrification, as described in [38], with minor modifications. The important details are summarised below. These instructions are empirical and not intended to limit the scope of use of these grids, but rather to provide a starting point for routine applications.

### Handling

The grids can be handled by sharp tweezers, and only by the rim to avoid damage to the foil. The integrity of the foil can be assessed by looking at the grid against bright white light in the background. The small-hole foil should appear blue in transmission illumination due to a plasmon resonance caused by the regular hole pattern. The grid bar side of the grid appears light yellow in reflected light, whereas the foil side is shiny and with a brown tint.

### Surface treatment

The grids need to be made hydrophilic prior to use, just like other commercially available supports. This can be achieved, for example, by using residual air glow discharge or plasma treatment (Table A.3). We use a Fischione 1070 plasma chamber with a 9:1 Ar:O${}_{2}$ gas mixture to expose the grids to plasma generated at 40 W power for 120–180 s. Whilst the exact parameters may differ depending on the machine, doubling the plasma exposure time compared to that used for UltrAuFoil grids is usually a good starting point for optimisation.

### Vitrification

Plasma-treated grids are used immediately (within 15 min or less) for vitrification. We usually apply the sample to the foil side of the grid, and blot either from the same side or both sides, but any variation is possible and works with these grids with appropriate blotting force. We typically use a manual plunger and tend to blot slightly longer than for a large hole foil (15 s rather than 10 s). For example, on a Vitrobot Mk. 4 we blot for 5 s without wait or drain time and blot force ‘10’, but this will vary by machine. On a Leica GP2 plunger we use 5 s blot time and no drain or wait. We operate all cryo-plunging instruments at 4 °C and 100% relative humidity to reduce evaporation from the thin water layers after blotting (Table A.4). Grids are always vitrified in liquid ethane, kept at 93 K, using a custom temperature controlled cryostat for the manual plunger or the Vitrobot [39].

### Data collection

To harness the full benefit of the closely packed holes on the HexAuFoil supports, we use automated data collection with aberration-free image shift (AFIS), fringe-free illumination, no specimen tilt, and a fast and efficient direct electron detector. We acquire only one exposure per hole, usually at magnifications corresponding to pixel sizes around 0.5–0.8 Å/pix on the detector in order to capture the whole hole in each micrograph. The illumination is set with a beam concentric with the hole and a beam diameter slightly larger than that of the hole [15], for example 500 nm beam for 300 nm holes, but sufficiently small so as not to expose neighbouring holes. Up to 700 nm beam diameter is acceptable for the 300 nm hole, 600 nm centre-to-centre distance hexagonal pattern, allowing for up to ±100 nm inaccuracy in the centring of each hole. Accurate calibration of the AFIS and the image shifts is essential to achieve such a high centring accuracy or better, and a fringe-free illumination alignment is required in most cases. Alternatively, the users may opt for a larger beam diameter (1μm), exposing and thus having to skip the neighbouring holes of each acquisition position, like was done in [21]. The pitch of the regular hole lattice can be used for low magnification calibration, for example to accurately measure the beam diameter, and for accurate image shift calibrations. As with UltrAuFoil grids, a separate grid with an amorphous foil (e.g. carbon or platinum/iridium) is needed for microscope alignments such as coma and astigmatism.

### Data processing

The edges of gold foil captured in each electron micrograph do not impede the cryo-EM data processing, and in fact provide a built-in magnification calibration using the gold lattice reflections (2.347 Å). The edges are also high-contrast fiducials for motion correction. For these small hole sizes, which eliminate electron beam induced movement of the vitreous ice, only whole-micrograph motion correction *without* subdivision into patches is necessary. In all other aspects, data processing does not diverge from the standard practices. Bayesian polishing [40] can still be used to confirm the absence of correlated particle movements within each hole, but is not necessary and does not significantly improve the resolution of the resulting reconstruction in the absence of significant movement other than stage drift. Final high-resolution reconstructions can be used to extrapolate the structure factors of the molecule to those of the undamaged structure, at zero electron dose, as described in [21].

## Appendix C Supplementary data

MMC S1 The supplementary file contains the mask designs for the HexAuFoil design described in the paper. The digitised data in the file/areas shown in colour (all closed polygons) in the data viewer will be clear/glass on the final masks. Each of the two layers (1 and 2) is a different mask, and layer 255 is the bounding box, which shows the extent of both masks. These designs are for 5 inch by 5 inch masks used to pattern a 100 mm diameter silicon wafer.

## References

[1] Russo C.J., Passmore L.A. (2016). Progress towards an optimal specimen support for electron cryomicroscopy. Curr. Opin. Struct. Biol..

[2] D’Imprima E., Kühlbrandt W. (2021). Current limitations to high-resolution structure determination by single-particle cryoEM. Q. Rev. Biophys..

[3] McMullan G., Faruqi A., Henderson R. (2016). Direct electron detectors. Methods Enzymol..

[4] Schorb M., Haberbosch I., Hagen W.J., Schwab Y., Mastronarde D.N. (2019). Software tools for automated transmission electron microscopy. Nature Methods.

[5] Cheng A., Negro C., Bruhn J.F., Rice W.J., Dallakyan S., Eng E.T., Waterman D.G., Potter C.S., Carragher B. (2021). Leginon: New features and applications. Prot. Sci..

[6] Marton L. (1934). Electron microscopy of biological objects. Nature.

[7] Bradley D.E. (1954). Evaporated carbon films for use in electron microscopy. Br. J. Appl. Phys..

[8] Baumeister W., Seredynski J. (1976). Preparation of perforated films with predeterminable hole size distributions. Micron.

[9] Fujiyoshi Y. (1998). The structural study of membrane proteins by electron crystallography. Adv. Biophys..

[10] Ghanim G.E., Fountain A.J., van Roon A.-M.M., Rangan R., Das R., Collins K., Nguyen T.H.D. (2021). Structure of human telomerase holoenzyme with bound telomeric DNA. Nature.

[11] Ermantraut E., Wohlfart K., Tichelaar W. (1998). Perforated support foils with pre-defined hole size, shape and arrangement. Ultramicroscopy.

[12] Quispe J., Damiano J., Mick S., Nackashi D., Fellmann D., Ajero T., Carragher B., Potter C. (2007). An improved holey carbon film for cryo-electron microscopy. Microsc. Microanal..

[13] Marr C., Benlekbir S., Rubinstein J. (2014). Fabrication of carbon films with ∼500 nm holes for cryo-EM with a direct detector device. J. Struct. Biol..

[14] Russo C.J., Passmore L.A. (2014). Ultrastable gold substrates for electron cryomicroscopy. Science.

[15] Russo C.J., Passmore L.A. (2016). Ultrastable gold substrates: Properties of a support for high-resolution electron cryomicroscopy of biological specimens. J. Struct. Biol..

[16] Yip K.M., Fischer N., Paknia E., Chari A., Stark H. (2020). Atomic-resolution protein structure determination by cryo-EM. Nature.

[17] Nakane T., Kotecha A., Sente A., McMullan G., Masiulis S., Brown P.M., Grigoras I.T., Malinauskaite L., Malinauskas T., Miehling J., Uchanski T., Yu L., Karia D., Pechnikova E.V., de Jong E., Keizer J., Bischoff M., McCormack J., Tiemeijer P., Hardwick S.W., Chirgadze D.Y., Murshudov G., Aricescu A.R., Scheres S.H. (2020). Single-particle cryo-EM at atomic resolution. Nature.

[18] Pawlak J., Cheng P., Shinozaki D., Cheng P., Jan G. (1987). X-Ray Microscopy.

[19] Li J., Stein D., McMullan C., Branton D., Aziz M.J., Golovchenko J.A. (2001). Ion-beam sculpting at nanometre length scales. Nature.

[20] Yoshioka C., Carragher B., Potter C.S. (2010). Cryomesh: A new substrate for cryo-electron microscopy. Microsc. Microanal..

[21] Naydenova K., Jia P., Russo C.J. (2020). Cryo-EM with sub-1 Å specimen movement. Science.

[22] Naydenova K., Muir K.W., Wu L.-F., Zhang Z., Coscia F., Peet M.J., Castro-Hartmann P., Qian P., Sader K., Dent K., Kimanius D., Sutherland J.D., Löwe J., Barford D., Russo C.J. (2021). Structure of the SARS-CoV-2 RNA-dependent RNA polymerase in the presence of favipiravir-RTP. Proc. Natl. Acad. Sci. USA.

[23] Gardiner A.T., Naydenova K., Castro-Hartmann P., Nguyen-Phan T.C., Russo C.J., Sader K., Hunter C.N., Cogdell R.J., Qian P. (2021). The 2.4 Å cryo-EM structure of a heptameric light-harvesting 2 complex reveals two carotenoid energy transfer pathways. Sci. Adv..

[24] Solak H.H., Dais C., Clube F. (2011). Displacement Talbot lithography: a new method for high-resolution patterning of large areas. Opt. Express.

[25] Jefimovs K., Romano L., Vila-Comamala J., Kagias M., Wang Z., Wang L., Dais C., Solak H., Stampanoni M., Hohle C.K. (2017). Advances in Patterning Materials and Processes XXXIV.

[26] Rangelow I.W. (2003). Critical tasks in high aspect ratio silicon dry etching for microelectromechanical systems. J. Vacuum Sci. Technol. A.

[27] Wu B., Kumar A., Pamarthy S. (2010). High aspect ratio silicon etch: A review. J. Appl. Phys..

[28] T. Overstolz, P. Clerc, W. Noell, M. Zickar, N. de Rooij, A clean wafer-scale chip-release process without dicing based on vapor phase etching, in: 17th IEEE International Conference on Micro Electro Mechanical Systems. Maastricht MEMS 2004 Technical Digest, 2004, pp. 717–720.

[29] Sari I., Zeimpekis I., Kraft M. (2010). A full wafer dicing free dry release process for MEMS devices. Procedia Eng..

[30] Chang J.-K., Fang H., Bower C.A., Song E., Yu X., Rogers J.A. (2017). Materials and processing approaches for foundry-compatible transient electronics. Proc. Natl. Acad. Sci. USA.

[31] Dewa A.S. (2004).

[32] Abadias G., Chason E., Keckes J., Sebastiani M., Thompson G.B., Barthel E., Doll G.L., Murray C.E., Stoessel C.H., Martinu L. (2018). Stress in thin films and coatings: Current status, challenges, and prospects. J. Vacuum Sci. Technol. A.

[33] Pu S.H., Holmes A.S., Yeatman E.M. (2013). Stress in electroplated gold on silicon substrates and its dependence on cathode agitation. Microelectron. Eng..

[34] Wei H., Dandey V.P., Zhang Z., Raczkowski A., Rice W.J., Carragher B., Potter C.S. (2018). Optimizing “self-wicking” nanowire grids. J. Struct. Biol..

[35] Naydenova K., Peet M.J., Russo C.J. (2019). Multifunctional graphene supports for electron cryomicroscopy. Proc. Natl. Acad. Sci. USA.

[36] D’Imprima E., Floris D., Joppe M., Sanchez R., Grininger M., Kühlbrandt W. (2019). Protein denaturation at the air-water interface and how to prevent it. ELife.

[37] Zheng L., Chen Y., Li N., Zhang J., Liu N., Liu J., Dang W., Deng B., Li Y., Gao X., Tan C., Yang Z., Xu S., Wang M., Yang H., Sun L., Cui Y., Wei X., Gao P., Wang H.-W., Peng H. (2020). Robust ultraclean atomically thin membranes for atomic-resolution electron microscopy. Nature Commun..

[38] Passmore L.A., Russo C.J. (2016). Specimen preparation for high-resolution cryo-EM. Methods Enzymol..

[39] Russo C.J., Scotcher S., Kyte M. (2016). A precision cryostat design for manual and semi-automated cryo-plunge instruments. Rev. Sci. Instrum..

[40] Zivanov J., Nakane T., Scheres S.H.W. (2019). A Bayesian approach to beam-induced motion correction in cryo-EM single-particle analysis. IUCrJ.

